# Breathing as an Input Modality in a Gameful Breathing Training App (Breeze 2): Development and Evaluation Study

**DOI:** 10.2196/39186

**Published:** 2022-08-16

**Authors:** Yanick Xavier Lukic, Gisbert Wilhelm Teepe, Elgar Fleisch, Tobias Kowatsch

**Affiliations:** 1 Centre for Digital Health Interventions Department of Management, Technology, and Economics ETH Zurich Zurich Switzerland; 2 Centre for Digital Health Interventions Institute of Technology Management University of St.Gallen St.Gallen Switzerland; 3 Institute for Implementation Science in Health Care University of Zurich Zurich Switzerland; 4 School of Medicine University of St.Gallen St.Gallen Switzerland

**Keywords:** breathing training, serious game, biofeedback, digital health, mobile health, mHealth, mobile phone, machine learning, deep learning, transfer learning, neural networks

## Abstract

**Background:**

Slow-paced breathing training can have positive effects on physiological and psychological well-being. Unfortunately, use statistics indicate that adherence to breathing training apps is low. Recent work suggests that gameful breathing training may help overcome this challenge.

**Objective:**

This study aimed to introduce and evaluate the gameful breathing training app Breeze 2 and its novel real-time breathing detection algorithm that enables the interactive components of the app.

**Methods:**

We developed the breathing detection algorithm by using deep transfer learning to detect inhalation, exhalation, and nonbreathing sounds (including silence). An additional heuristic prolongs detected exhalations to stabilize the algorithm’s predictions. We evaluated Breeze 2 with 30 participants (women: n=14, 47%; age: mean 29.77, SD 7.33 years). Participants performed breathing training with Breeze 2 in 2 sessions with and without headphones. They answered questions regarding user engagement (User Engagement Scale Short Form [UES-SF]), perceived effectiveness (PE), perceived relaxation effectiveness, and perceived breathing detection accuracy. We used Wilcoxon signed-rank tests to compare the UES-SF, PE, and perceived relaxation effectiveness scores with neutral scores. Furthermore, we correlated perceived breathing detection accuracy with actual multi-class balanced accuracy to determine whether participants could perceive the actual breathing detection performance. We also conducted a repeated-measure ANOVA to investigate breathing detection differences in balanced accuracy with and without the heuristic and when classifying data captured from headphones and smartphone microphones. The analysis controlled for potential between-subject effects of the participants’ sex.

**Results:**

Our results show scores that were significantly higher than neutral scores for the UES-SF (*W*=459; *P*<.001), PE (*W*=465; *P*<.001), and perceived relaxation effectiveness (*W*=358; *P*<.001). Perceived breathing detection accuracy correlated significantly with the actual multi-class balanced accuracy (*r*=0.51; *P*<.001). Furthermore, we found that the heuristic significantly improved the breathing detection balanced accuracy (*F*_1,25_=6.23; *P*=.02) and that detection performed better on data captured from smartphone microphones than than on data from headphones (*F*_1,25_=17.61; *P*<.001). We did not observe any significant between-subject effects of sex. Breathing detection without the heuristic reached a multi-class balanced accuracy of 74% on the collected audio recordings.

**Conclusions:**

Most participants (28/30, 93%) perceived Breeze 2 as engaging and effective. Furthermore, breathing detection worked well for most participants, as indicated by the perceived detection accuracy and actual detection accuracy. In future work, we aim to use the collected breathing sounds to improve breathing detection with regard to its stability and performance. We also plan to use Breeze 2 as an intervention tool in various studies targeting the prevention and management of noncommunicable diseases.

## Introduction

### Background

Noncommunicable diseases (NCDs) are a substantial global health and economic burden [1-3]. Slow-paced breathing training is positively associated with physiological [4-6] and psychological [7-9] well-being. Thus, breathing training can play a role in interventions targeting NCDs. For example, slow-paced breathing training may induce relaxation and help counteract stress [8]. It can also improve cardiac functioning [10], potentially enabling improved treatment of cardiovascular diseases, the leading cause of death worldwide [1]. Furthermore, it can strengthen respiratory muscles, rendering it relevant for the treatment of respiratory diseases such as asthma [4] and chronic obstructive pulmonary disease [11].

Slow-paced breathing training generally aims at guiding people to breathe with 5.5 to 6 breaths per minute (BPM) [6]. People may be able to maximize their personal effects by adjusting the BPM. For example, an untrained person may achieve better results by breathing with >6 BPM, whereas a well-trained person may want to breathe with <6 BPM. Nevertheless, 6 BPM are generally used as this appears to work well for most people, which results in 1 complete breathing cycle every 10 seconds. A breathing cycle consists of an inhalation, an exhalation, and up to 2 pauses in between. The duration of these individual phases is an area of active research. Investigating these separate phases is relevant as inhalation is associated with the sympathetic nervous system by inhibiting vagal outflow, and exhalation is associated with the parasympathetic nervous system by restoring vagal outflow [12,13].

Consequently, breathing patterns typically use equal inhalation and exhalation durations to balance the sympathetic and parasympathetic activity or prolonged exhalations to emphasize the parasympathetic nervous system. Although both approaches effectively induce relaxation [14], related work argues that a prolonged exhalation achieves more substantial relaxation effects. In contrast, other related work has found equal durations of inhalation and exhalation phases best suited to attain psychophysiological coherence [12,13,15].

The positive effects of breathing training have sparked the development of various breathing guidance apps [16]. Nevertheless, although these apps receive much attention and are downloaded by many users, the use statistics show that adherence is low [16,17]. The problem of nonadherence and lacking engagement is present in various domains, and different works hypothesize gamification as a potential solution [18,19]. In addition, for breathing training, various mobile [20-22], desktop [23], and virtual reality [24,25] applications that use some gameful elements have been conceptualized and developed. However, many apps are not interactive and do not include biofeedback during training, which is surprising as research indicates increased effectiveness of breathing training when biofeedback is used [26-29].

Nevertheless, breathing training apps exist that include biofeedback mechanisms based on heart rate variability (HRV) [30] or breathing [21-23,31]. Although HRV-based biofeedback offers feedback on the biosignal often targeted by breathing training [32], it only provides deferred feedback. It is also challenging to measure HRV without additional hardware. Thus, HRV biofeedback is neither scalable nor well suited as sole input for a gameful experience that requires feedback loops with latencies of <1 second.

By contrast, breathing-based biofeedback can be instantaneous and is the direct signal that the guidance in breathing training apps tries to change to then affect HRV. However, approaches using breathing-based biofeedback are so far limited to breathing training in controlled environments [21] and early prototypes [22]. One of the first apps to go in this direction was Breeze (Centre for Digital Health Interventions) [21]. The first version of Breeze featured a single environment where users accelerate a sailboat by correctly following a fixed breathing pattern. The effectiveness of Breeze in increasing HRV in individuals was shown in the laboratory [33].

Furthermore, Lukic et al [33] evaluated the effect of Breeze’s visualization and visual breathing training guidance on participants’ intrinsic experiential value. The results showed a significant increase in intrinsic experiential value when the gameful visualization was used compared with a standard guidance visualization while maintaining the same perceived effectiveness (PE) [34]. Nevertheless, investigations showed that the breathing phase detector used, enabling interactivity in Breeze, was very prone to noise and differences in individuals’ breathing sounds and was overfitted on the data set used [21]. Research by Islam et al [35] extended the idea of breathing phase detection from breathing training–specific breathing to regular breathing. They focused on monitoring and diagnosis as measuring breathing phases in normal breathing has been motivated for diagnostic purposes [36,37]. Generally, monitoring and diagnosis are popular areas of research regarding breathing detection. Although Islam et al [35] aimed to monitor breathing phases during rest, others tried to detect breathing rates during sleep [38] and physical exercise [39] through smartphone and headphone microphones. However, as breathing training guides breathing, it does not make sense to investigate breathing phase durations and breathing rates during this time with diagnostic intentions. Breathing detection in the context of breathing training aims to provide interactive feedback to users to increase engagement and give them a tangible assessment of their performance.

### Objectives

This paper introduces Breeze 2, which has several new features, an improved appearance, and a novel breathing phase detection algorithm. We designed Breeze 2 as a stand-alone training and an intervention component in multicomponent interventions. Breeze 2 adds a slide-based tutorial to introduce users to breathing training and biofeedback mechanics. Furthermore, it allows for the setting of specific training parameters (ie, training duration and breathing pattern) according to the user’s breathing training experience. It also adds procedural generation of the visual biofeedback environment so users always have a slightly different experience when performing breathing training. Consequently, this study has the following objectives: (1) to provide a detailed description of Breeze 2, a revised gameful breathing training app for smartphones; (2) to introduce and evaluate a novel real-time breathing phase detection approach based on deep transfer learning and an additional heuristic that prolongs detected exhalations to stabilize the algorithm’s predictions; and (3) to evaluate perceived engagement, PE, and breathing detection performance in a laboratory setting with 30 participants.

## Methods

### Design and Implementation

#### Overview

Breeze 2 provides visual breathing guidance through gameful visualizations. Furthermore, it incorporates interactive components that aim to make the training more engaging and provide valuable feedback to users on their breathing training performance. We outline the details of the revised user interface design and breathing detection algorithm of Breeze 2 in the following sections.

#### Concept and Design

##### Overview

In contrast to many other breathing training systems, Breeze 2 does not rely on abstract shapes but uses a tangible setting that allows for the design of the interactive components in a relatable way. A sailboat that continuously moves forward slowly guides the breathing training. Depending on the user’s ability to match the guiding breathing pattern, the exhalation triggers stronger winds in the experience, and the sailboat accelerates. This way, the sailboat travels a larger distance over the duration of the training, which allows for the use of the traveled distance as a condensed measure of training performance aside from more precise measurements such as the timing accuracies on exhalations.

In addition to the breathing training itself, Breeze 2 also offers a tutorial, adjustable training parameters, and procedural generation to vary the shown environment for every breathing training session.

##### Tutorial

**Figure 1:**
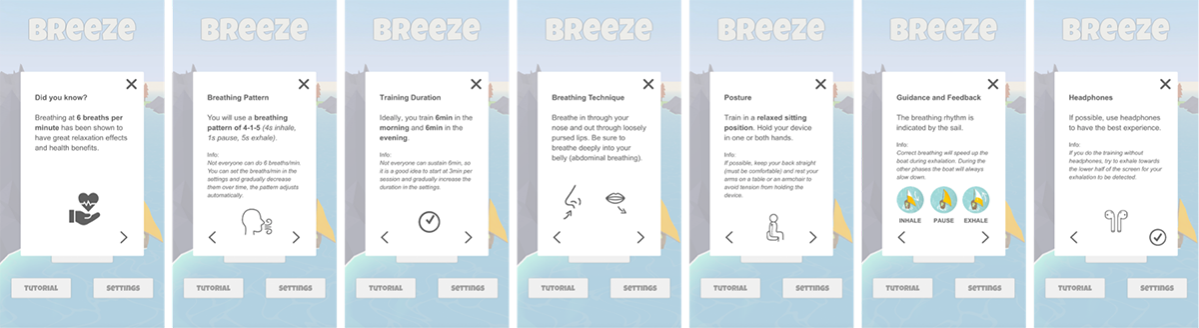
Slide-based tutorial as implemented in Breeze 2. It provides high-level information on the benefits of slow-paced breathing training and its biofeedback mechanics.

Breeze 2 uses a simple slide-based tutorial (Figure 1) that quickly introduces the user to the benefits of slow-paced breathing training and briefly explains the guidance and interaction components. When a user opens Breeze 2 for the first time, the start button on the home screen is disabled. Once the user has completed the tutorial, the start button is enabled, and the user can start a training session.

##### Training Parameters

**Figure 2:**
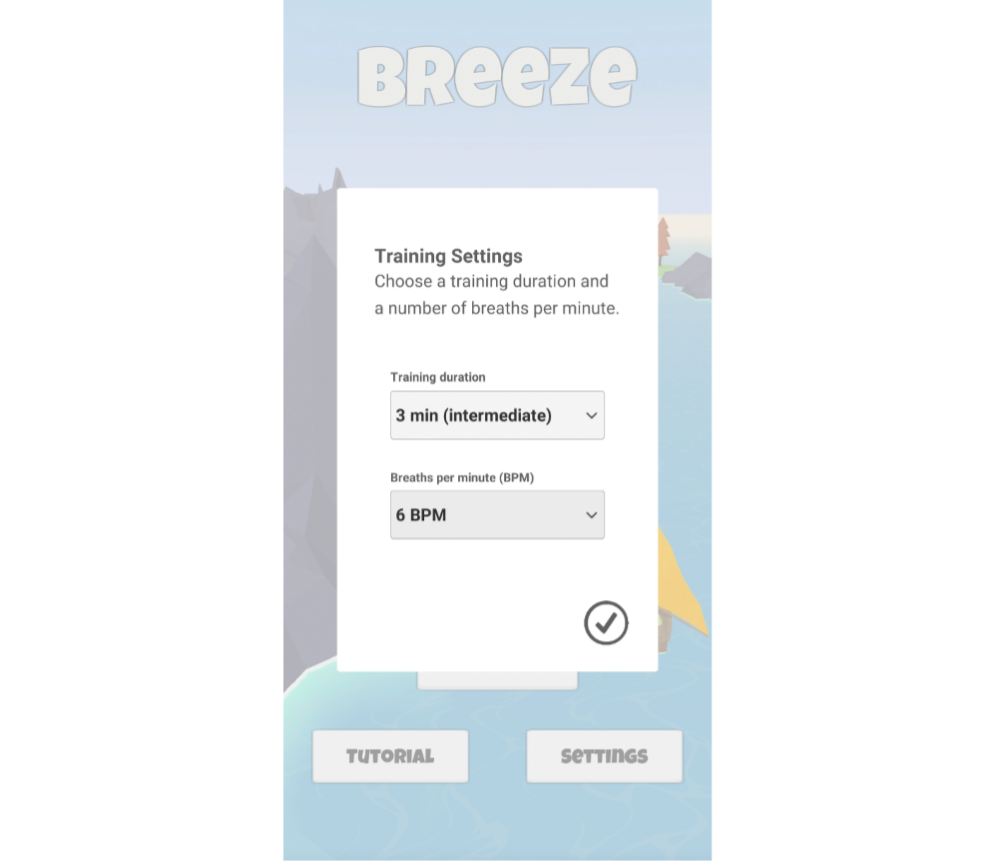
Settings screen where the users can set the training duration and the breaths per minute according to their preferences.

Breathing training mainly consists of 2 parameters: the training duration and the breathing pattern. Users can adapt both according to their preferences. Breeze 2 supports this process by labeling the possible durations according to their required level of breathing training experience. We chose the breathing training parameters based on feedback from medical professionals working with biofeedback-guided breathing exercises as patient treatment. Regarding training durations, the user can choose between *2 (beginner)*, *3 (intermediate)*, and *5 (expert)* minutes. The breathing patterns in breathing training usually take the form of *inhalation-pause-exhalation-pause*. Breeze 2 uses the pattern *4-1-5-0* and adjusts the inhalation and exhalation duration linearly to match the desired BPM. For example, if 6 BPM is selected, the breathing pattern follows 4, 1, 5, and 0 seconds of inhalation; first pause; exhalation; and second pause. However, if 7 BPM is selected, the pattern follows 3.37, 1, 4.21, and 0 for the 4 phases. As a standard selection, we used 6 BPM. Figure 2 illustrates Breeze’s settings screen.

##### Voice Commands

Breeze 2 allows for the enabling of voice commands to start and end breathing training. We implemented this feature to enable future studies using Breeze to gather and analyze voice features regarding the studies’ outcomes. If a user speaks for a specified time, Breeze 2 approves the command. We can configure the content and expected durations of commands according to the studies’ needs. Consequently, Breeze 2 does not check whether the user said the correct words but that they said something. This way, it is less error-prone and allows users to speak more naturally, with the caveat that it is required to trust the user to speak the displayed words. We used a pretrained Yet Another Mobile Network (YAMNet) model for the necessary voice detection [40].

##### Interaction During Training

**Figure 3:**
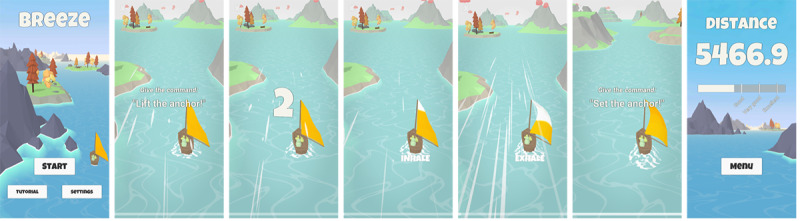
An entire session of Breeze 2 (from left to right): start screen, starting voice commands, countdown, inhalation phase, exhalation phase, ending voice commands, and final screen.

Users can initiate a breathing training session from the start menu. When the training starts, the view changes to the training mode. Initially, users see a sailboat floating on a river from behind. The sailboat first stands still. For the next step, the users need to read 3 voice commands aloud, after which a countdown starts. At the end of the countdown, the guidance breathing pattern starts. The BPM parameter the user sets determines how long the individual phases are in seconds. An animation on the sail of the sailboat represents the separate phases. During the first 5 breathing cycles, Breeze 2 also indicates the breathing phases through an additional text label below the sailboat. In the beginning, the sailboat moves forward at a slow constant speed. The users must then adapt their breathing to the breathing pattern by following the guidance system. The more accurately the users follow the pattern, the more strongly the sailboat accelerates during the exhalation phase. A correctly timed exhalation triggers a wind animation that propels the sailboat forward. During the inhalation and pause phases, the sailboat’s speed slowly decreases until it reaches the constant base speed. Once users complete a training session, they again speak 3 voice commands aloud. After that, the users see a new screen showing the reached distance and the breathing accuracy over the completed session. Figure 3 depicts a complete training session. A screen recording of a complete session without voice commands can be found in Multimedia Appendix 1.

##### Procedural Generation

**Figure 4:**
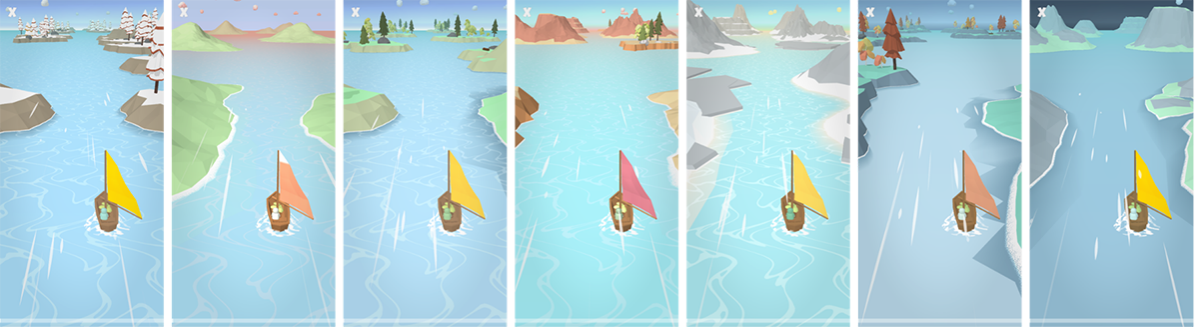
A selection of procedurally generated landscapes during breathing training sessions.

As users should perform breathing training regularly, it is essential to provide visual variety every time to support long-term adherence. Breeze 2 uses simple procedural generation that varies the environment’s appearance and lighting during training sessions to achieve this visual variety. The procedural generation works with predefined configurations that include groups of 3D models and different coloring and lighting schemes. We handcrafted these configurations to ensure that they fit together. Every session, the app randomly chooses one of the configurations. Subsequently, the procedural generation places island models assigned to this configuration along the travel path of the sailboat at random locations. Furthermore, this configuration’s coloring and lighting scheme are chosen and applied to the scene. Figure 4 illustrates such generated scenes, including islands, coloring schemes, and landscapes.

##### Background Sounds

If the users use Breeze 2 without headphones, it does not have any sound to not interfere with breathing detection. Otherwise, Breeze 2 plays a peaceful background sound during the training session. It combines soft water sounds with subtle animal sounds such as birds. The background sound is audible in the screen recording in Multimedia Appendix 1.

#### Stand-alone and Intervention Component

Breeze 2 can be used either as a stand-alone breathing intervention or as an intervention component for multicomponent interventions. For the former, a start screen allows the users to set training parameters via the settings menu (Figure 2) and a simple slide-based tutorial (Figure 1). When built as an intervention component for a multicomponent intervention, training parameters can also be handed over as parameters to Breeze 2, and the training may start right away. The handing over of parameters is useful if the multicomponent intervention (eg, a smartphone-based holistic lifestyle intervention) already features a tutorial and the possibility to choose training parameters (eg, via chatbot).

#### Implementation

We used the Unity real-time development platform (version *2020.3.4f1*; Unity Technologies) to implement Breeze 2. All 3D models were custom creations or acquired through the Unity Asset Store. For 3D model creation and modification, we used the 3D modeling software Blender (Blender Foundation). The background sound was downloaded from Freesound [41] and was available under the Creative Commons Zero license.

### Real-time Breathing Detection

#### Overview

The aim of breathing detection for interactive breathing training is to detect inhalation and exhalation phases as fast as possible to enable real-time feedback. Consequently, the detection algorithm must distinguish these 2 phases and all nonbreathing sounds. Previous work that tried to detect breathing phases during breathing training [21] and natural breathing at rest [35] used preceding breathing detection gates that check inputs for breathing sounds before passing them to the model that classifies only breathing-related classes. Shih et al [21] tried to detect breathing in close to real time and used a breathing gate that works on 1-second clips. Aside from inhalations and exhalations, they also tried to detect breathing pauses. However, as they applied a sequence model and wanted to account for clips that included different phases, they split pause phases into inhalation-pause and exhalation-pause.

Islam et al [35] focused on breathing monitoring and diagnosis and, thus, used a 1-minute breathing gate. They also made the simplifying assumption that breathing is continuous and has no pauses. This focus and assumption allowed them to reduce the problem to a 2-class problem for their primary model with the classes inhalation and exhalation.

We did not apply a sequence model and aimed for real-time predictions. Thus, we could not work with input durations of 1 minute. Furthermore, we argue that a single model approach can be beneficial as the primary model then does not only come into contact with a limited domain. Consequently, we used only 1 model and defined the problem as a 3-class problem with the classes inhalation, exhalation, and nonbreathing sounds (including silence).

Similar to Shih et al [21], this work focuses on applying a breathing detector in breathing training guided by an app running on a smartphone. This comes with a caveat as, when detecting exhalations using a smartphone’s microphone, it is essential to distinguish between detecting the exhalation from sound alone and the airflow itself. Users may exhale toward the device during training, leading to disturbances in the audio recording usually produced by wind. Identifying these disturbances is especially relevant if slow-paced breathing is combined with pursed-lip breathing as the air stream is becoming more focused this way. Therefore, the model should still detect the resulting disturbance sounds as exhalations resulting in 2 subtypes of the exhalation class, which we call acoustic and airflow exhalations in this paper. However, the model should assign samples from both subtypes to the exhalation class regardless of whether they are acoustic or airflow exhalations.

#### Data Set

We formed the data set used for training, validation, and preliminary testing from 3 separate data sets. The first consists of acoustic breathing sounds, the second consists of exhalation-generated airflow disturbance sounds, and the third consistis of environmental sounds.

We used a subset of the data set from Shih et al [21] for acoustic breathing sounds. We only used the recordings produced by the RØDE NT1000 microphone, which had the best quality. Furthermore, we manually selected only recordings that contained audible breathing and little or no constant background noise, which resulted in audio data from 20 participants. As breathing training is often performed by inhaling through the nose and exhaling through the mouth, we only included these sounds for the breathing data set. Data from the first 80% (16/20) of the participants served as training and validation data. The validation set contained the last 3 breathing cycles by a participant, whereas we used the remaining breathing cycles for training. Data from the remaining 20% (4/20) of the participants served as test data that we used to ensure model testing on only data from unseen individuals.

As the data set from Shih et al [21] only contains acoustic breathing sounds, we recorded new data for exhalations that produce disturbances in the recording through airflow. Given that these disturbance sounds are the same as those produced by wind hitting the microphone’s membrane, they are mostly independent of the individual’s breathing sound. The smartphone used has a more significant influence as the microphone’s position and the device’s overall architecture influence how much air reaches the microphone’s membrane. Consequently, a male and a female participant performed three 2-minute breathing training sessions. The 2 participants used different smartphones without headphones for the training sessions. Both participants exhaled toward the device during training. The exhalation sounds were then manually extracted from the resulting recordings. To ensure that the airflow sounds were independent of the individual, we only included the samples produced by the male participant in the training and validation sets and used the samples from the female participant in the test set.

For nonbreathing sounds, we used the data set ESC-50 [42], which entails 50 classes of environmental sounds. Every recording is 5 seconds long, with 40 recordings per class. We excluded all breathing sounds from the data set and used folds 1, 2, and 3 for the training, validation, and test set, respectively. We also used nonbreathing sounds and silence from the breathing sound data set from Shih et al [21]. They were distributed in the same way as the breathing sounds in the training, validation, and test sets. We used these nonbreathing sounds and silence to ensure the model did not use the environmental characteristics of the recordings to distinguish between breathing and nonbreathing sounds.

All recordings in the data set were then cut into 0.195-second–long nonoverlapping clips. Table 1 describes the resulting composition of the data set.

**Table 1:** Data set composition used for training, validation, and the testing of the developed model.

Class	Samples, n		
	Training	Validation	Testing
Exhalation (acoustic)	4574	941	753
Exhalation (airflow)	418	82	455
Inhalation	2470	478	663
Nonbreathing (ESC-50 data set)	9800	9800	9800
Nonbreathing (laboratory)	1952	1952	552

#### Transfer Learning Approach

##### Overview

We used a pretrained YAMNet [40] model as the basis for transfer learning. YAMNet is a convolutional neural network based on the MobileNetV1 [43] architecture trained on the AudioSet data set [44] to classify 521 classes. Transfer learning refers to using a pretrained model or relevant parts of it and fine-tuning it on a related problem [45].

##### Preprocessing

The audio samples were preprocessed to fit the YAMNet requirements. Specifically, we resampled the audio to 16 kHz mono. Here, we introduced a step specific to our problem. YAMNet uses a minimum of 15,600 data points as input, which corresponds to 0.975 seconds (internally, it works with 0.96-second patches but requires additional samples to compute the final Short-time Fourier transform window [40]). However, it is questionable whether 1 second is fast enough for real-time feedback that should be perceived as immediate. Research in touch-based systems indicates that commercial touch screens yield latencies of up to 200 ms [46] and that perceivable latency lies between 2 and 100 ms [47]. To the best of our knowledge, no such research exists for breathing inputs. We hypothesize that the perceived latency in breathing-based systems is not as sensitive as in touch-based systems.

Consequently, we aimed for an input size of >100 ms but still significantly <1 second to ensure that the input contained enough information but could still provide feedback that users may perceive as immediate. We decided to use 0.195 seconds as input size, corresponding to 3120 samples and one-fifth of a YAMNet input. We then concatenated this snippet to arrive at the total input for YAMNet. Not just padding the signal with some constant value ensures that inputs containing distinct sound sources are as different as possible from, for example, quiet environments. We then calculated a mel spectrogram with a window and hop size of 25 and 10 ms, respectively. The mel spectrogram consisted of 64 mel bins covering the range of 125 to 7500 Hz. Finally, we calculated the log mel spectrogram by calculating log(*S*+0.001), where *S* is the mel spectrogram.

##### Feasibility Check

**Figure 5:**
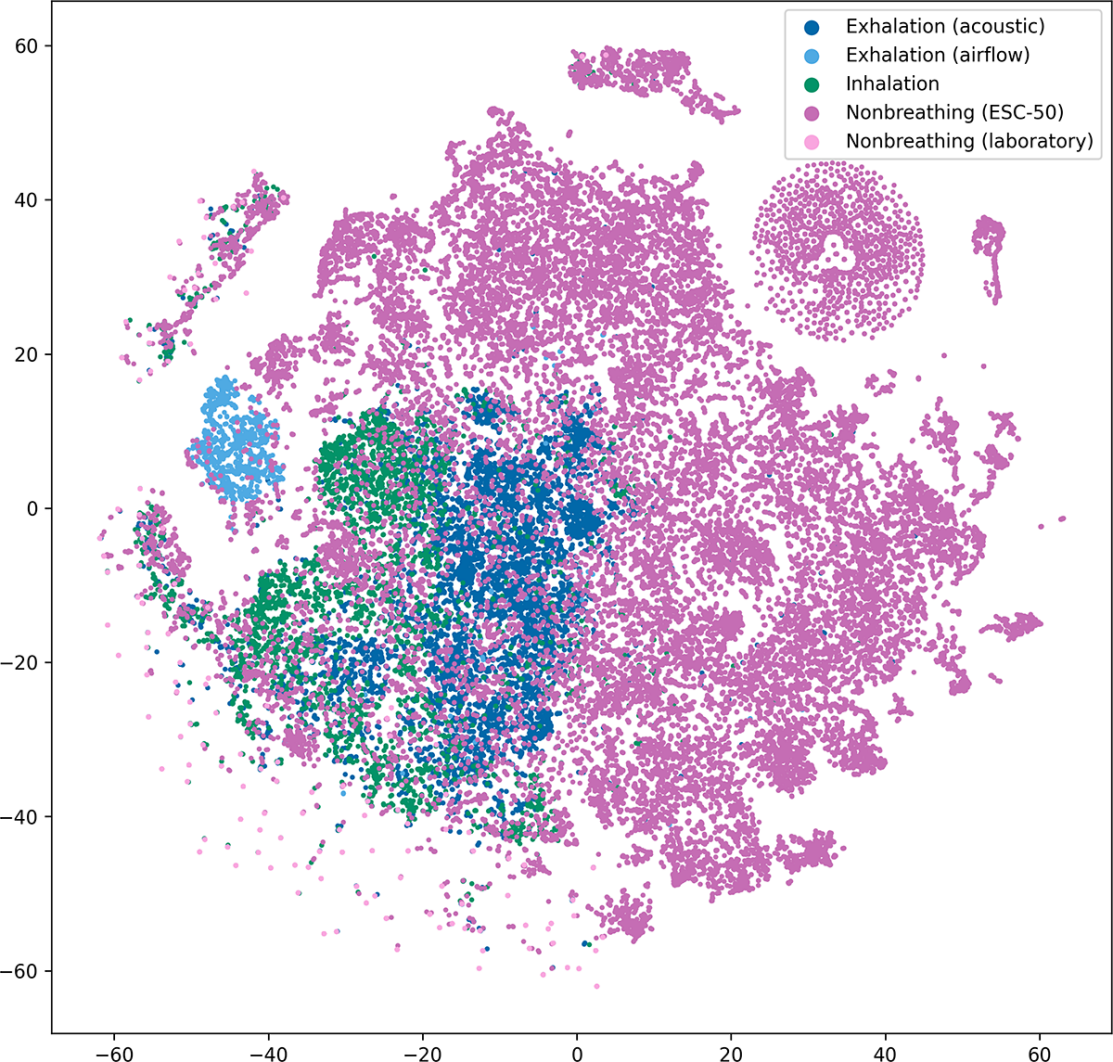
Visualized Yet Another Mobile Network (YAMNet) embeddings for the complete data set. We used t-distributed stochastic neighbor embedding to reduce the high dimension of the embeddings. ESC-50: Dataset for Environmental Sound Classification.

To assess whether the embeddings of YAMNet captured features that allowed for distinguishing between inhalation, exhalation, and nonbreathing sounds, we used the *t*-distributed stochastic neighbor embedding method [48]. We calculated embeddings for all samples in the data set, resulting in 1024-dimensional embeddings that we then reduced to 2D embeddings using *t*-distributed stochastic neighbor embedding (with Euclidean metric). We then visualized the 2D embeddings in a scatter plot and manually inspected these representations (Figure 5). We observed that the airflow exhalation samples clustered separately from the acoustic breathing sounds. In addition, airflow exhalations clustered together regardless of person and device. The acoustic exhalations also clustered together but partially overlapped with the inhalations. The visualization also showed that nonbreathing sounds formed various clusters among themselves as the ESC-50 data set contained different types of sounds. The samples from the laboratory containing nonbreathing sounds were also spread across a wide range but separated quite clearly from breathing sounds while partly overlapping with various ESC-50 clusters.

##### Training

For transfer learning, we used the 1024-dimensional embeddings generated by YAMNet and fed them into a small neural network consisting of 2 fully connected layers with 32 and 3 units. The first layer applied the swish [49] activation function, and the output layer applied the softmax function. We trained the algorithm using mini-batch gradient descent with the Adam optimizer and categorical cross-entropy as loss function. Mini-batch size was set to 32. Our manual testing showed that the algorithm usually started to overfit on the training set after 5 to 10 epochs. We then used early stopping with patience of 10 epochs and restored the best weights according to the lowest loss reached on the validation set. Even though the used data set was strongly imbalanced, we did not use any balancing approaches as there is more diversity in nonbreathing sounds than in exhalations and inhalations. This way, we wanted to discourage false positives on breathing sounds. Otherwise, the detector may yield problems in not perfectly quiet environments. Using this transfer learning approach, we created 1000 models and chose the 3 that reached the lowest loss on the validation set to be combined as an ensemble. The ensemble applied soft voting with equal model weights (the class with the maximum sum of probabilities is chosen). This was done to slightly increase the performance and stability of the model’s predictions.

##### Evaluation

**Figure 6:**
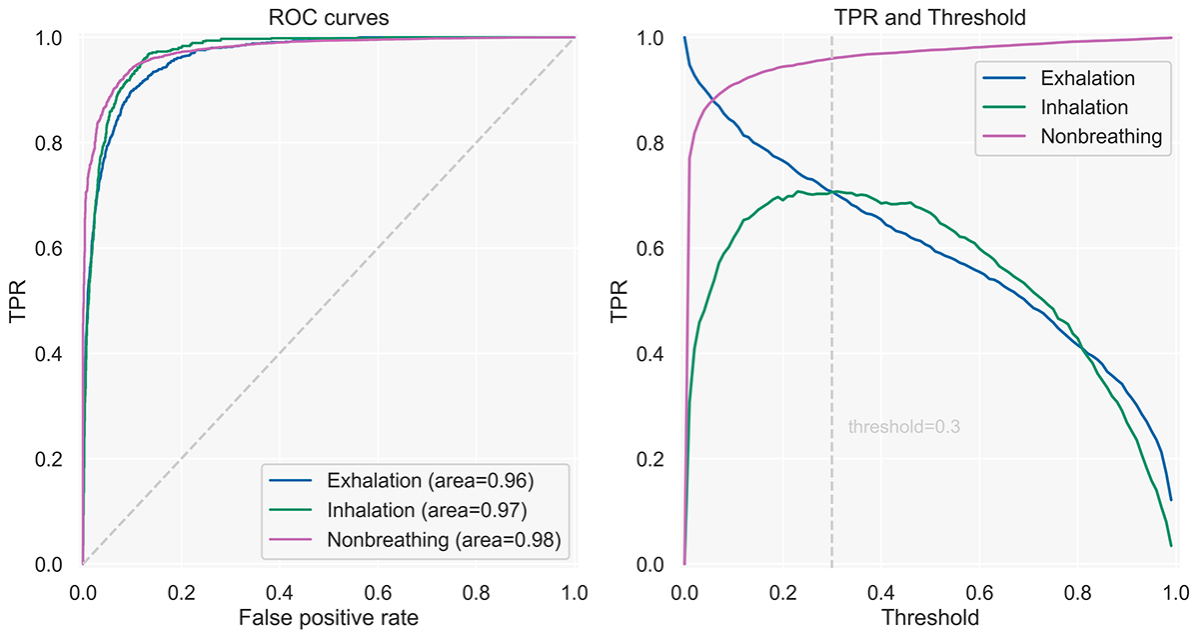
The left diagram depicts the model’s ROC curves for exhalation, inhalation, and nonbreathing sounds on the test set. The right chart shows the TPR for all classes when applying different thresholds for the breathing classes. We used the same threshold for inhalations and exhalations but applied it first to exhalations. We used the visual inspection of the right chart to determine a suitable threshold for the breathing classes. ROC: receiver operating characteristic; TPR: true positive rate.

To evaluate the model, we used the unseen test set. We investigated the receiver operating characteristic (ROC) curves; confusion matrix; and the precision, recall/sensitivity, specificity, *F*_1_ score, and balanced accuracy metrics. The ROC curves (Figure 6) yielded areas under the curve of 0.96, 0.97, and 0.98 for exhalation, inhalation, and nonbreathing sounds, respectively, indicating good discrimination capacity between all classes.

**Figure 7:**
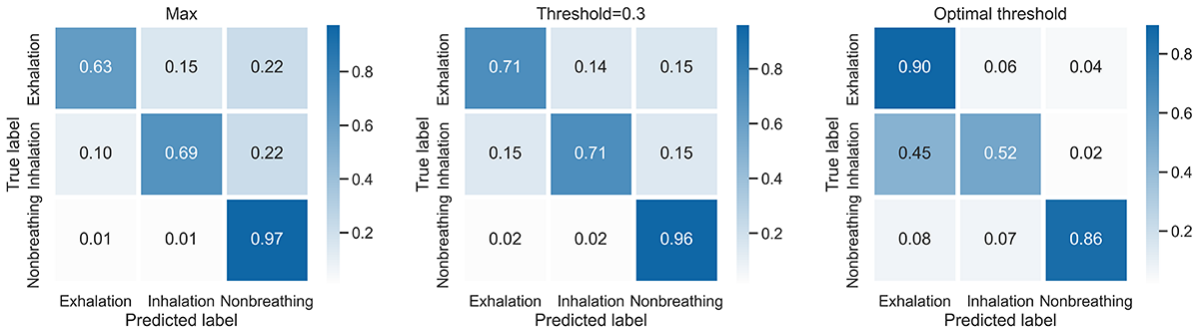
Confusion matrices showing the model results on the test data set applying 3 different thresholds for the breathing classes. From left to right: max (the class with the highest prediction probability is selected), threshold=0.3 (the threshold of 0.3 is applied first to exhalation and then to inhalation), and optimal threshold (different optimal thresholds for exhalation and inhalation are applied, as determined by the Youden J statistic).

To identify thresholds for exhalation and inhalation detection, we applied 2 approaches. First, we calculated the optimal thresholds for inhalation and exhalation individually by selecting the threshold that yielded the highest Youden *J* statistic [50]. Second, we plotted the true positive rates for several thresholds and established an appropriate threshold via visual inspection that yielded a balance between the 3 classes (Figure 6). We applied the threshold first for exhalation and then for inhalation and, if they did not apply, the model yielded nonbreathing. We found the threshold of 0.3 to strike a reasonable balance between the 3 classes. Figure 7 shows the confusion matrices for the standard threshold (maximum probability), the optimal thresholds, and the threshold of 0.3 for the test. We concluded that the threshold of 0.3 reached a better balance and, thus, discrimination among classes.

**Figure 8:**
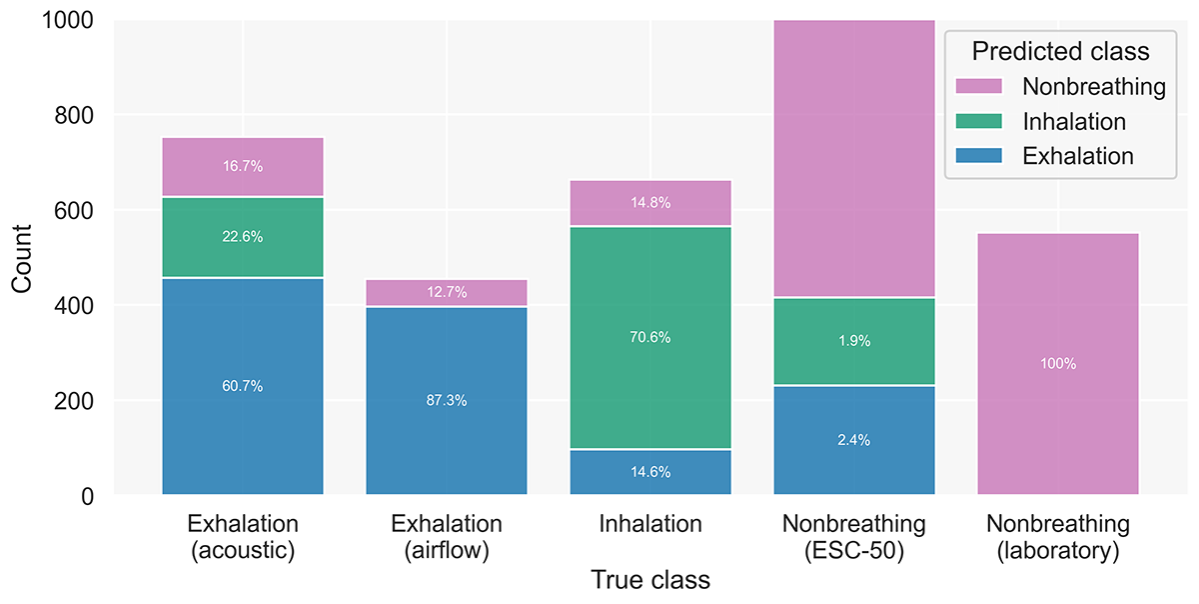
Histogram showing test set classifications by the model using a 0.3 threshold for breathing sounds. We split the data according to the subsets. As the ESC-50 subset of the nonbreathing sounds is substantially larger (9800 samples) than the other subsets, we cut off the diagram at 1000 samples. ESC-50: Dataset for Environmental Sound Classification.

Consequently, we chose this threshold for further evaluation. The precision, recall/sensitivity, specificity *F*_1_ score, and balanced accuracy metrics for this model are provided in Table 2. They show that the model best detects nonbreathing sounds, the most dominant class in the training set. The confusion matrices also show that the model more often misclassified exhalation and inhalation samples as nonbreathing sounds than as the wrong breathing phase. To gain further insights regarding the correct and incorrect classifications, we visualized test set classifications for all subclasses of the 3 main classes (Figure 8). The figure indicates that acoustic exhalations are similarly often misclassified as inhalations and nonbreathing sounds, whereas airflow exhalations are only misclassified as nonbreathing sounds. Inhalations yield a similar result as acoustic exhalations. For nonbreathing sounds, the samples from the laboratory appear to be easily distinguishable by the model. Sound samples from ESC-50 yield some misclassifications, with most being exhalations. Nevertheless, the misclassifications for nonbreathing sounds are only a small portion of all samples of this class.

**Table 2:** Performance metrics of the model using a threshold of 0.3 for the breathing classes on the data from the test set.

Class	Precision	Recall/sensitivity	Specificity	*F*_1_ score	Balanced accuracy
Exhalation	0.72	0.71	0.97	0.71	0.84
Inhalation	0.57	0.71	0.97	0.63	0.84
Nonbreathing	0.97	0.96	0.85	0.97	0.90
Average	0.75	0.79	0.93	0.77	0.86

#### Model Inference Time Measurement

We conducted a basic performance measurement of the resulting model on 3 smartphones. The main objective was to verify that the model could perform inference in <0.195 seconds, which corresponds to the input duration of the audio signal. As it can be expected that more powerful devices allow for faster inference, we focused on low- to midrange Android smartphones from different device manufacturers. We used the TensorFlow (Google Brain Team) Android benchmark app [51] to measure the performance of our model after conversion to a TensorFlow Lite model. The benchmark app simulates the model’s execution within an actual Android app. Thereby, it is ensured that Android’s scheduler treats the thread and process priorities of the model inference like those of a foreground app. We ran inference time measurements on a Samsung Galaxy S10e, a OnePlus 6, and a Huawei P30 Lite. All devices were factory reset before the benchmark app was installed. Furthermore, auto-lock was disabled to ensure that the devices did not switch to low-power mode during measurements. No hardware acceleration was used (ie, the use of a graphics processing unit, the NNAPI, the XNNPACK, and Hexagon was disabled in the benchmark app). We performed the measurements for 1, 2, and 4 central processing unit threads. For every device and thread configuration, we ran 100 inferences with 1 warm-up run. As the model was continuously running in the target use case, the warm-up and initialization times were neglectable and, thus, not reported. Table 3 lists the average measurements and their SDs. On the Samsung Galaxy S10e, measurements became unstable when using >2 central processing unit threads.

**Table 3:** Inference timings for the model on a small battery of Android smartphones.

Device	Inference time (*μ*; ms), mean (SD)		
	1 CPU^a^ thread	2 CPU threads	4 CPU threads
Samsung Galaxy S10e	7.71 (0.10)	5.44 (0.09)	7.05 (1.26)
OnePlus 6	15.37 (0.02)	9.39 (0.06)	6.42 (0.10)
Huawei P30 Lite	24.17 (0.13)	15.19 (0.99)	11.61 (1.32)

^a^CPU: central processing unit.

Consequently, we used 2 threads for model inferences in Breeze 2. The measurements showed that the model can make an inference on all tested smartphones below the input size of 1 sample (0.195 seconds). Therefore, it should be able to monitor an incoming audio stream seamlessly.

#### Implementation in Breeze 2

As the feedback mechanism focuses mainly on the exhalation, we used an additional heuristic for exhalation detection. For this heuristic, we exploited the high precision of exhalation detection and the fact that detection runs every frame (approximately 30 times per second). If an exhalation was detected, we used a delay of 300 ms, during which the heuristic set the detected phase to exhalation. Every time the model detected an exhalation, the heuristic reset the delay. This way, once an exhalation was detected, the user could receive an immediate reaction, which the heuristic maintained for at least 300 ms. We used this maintaining of detected exhalations to counter the expected moderate detection performance on exhalations when deploying the model in settings with higher background noise levels. Furthermore, it prevented too abrupt changes between sailboat acceleration and deceleration. Consequently, the heuristic was specific to the feedback loop used of the sailboat accelerating during correct exhalations.

### User Study

We aimed to evaluate this new iteration of Breeze and evaluate the breathing detection algorithm on new and realistic data through a laboratory study.

#### Participants

We recruited 30 participants (women: n=14, 47%; age: mean 29.77, SD 7.33 years). As our main interest was to collect breathing sounds and explore how certain aspects of Breeze 2 were perceived and how well breathing detection worked, the nature of the sample was not crucial. Consequently, we recruited participants mainly from ETH Zürich, but participation was open to all interested parties. However, participants had to be aged ≥18 years and not pregnant. Furthermore, they were required not to be taking any medication to treat depression, anxiety, or the main symptoms of mood disorders (such as low mood) and not to have any respiratory diseases such as asthma or chronic obstructive pulmonary disease. We aimed to balance female and male participants to account for potential differences in breathing sounds that may occur owing to physiological differences in respiratory function [52].

#### Materials

We measured *user engagement* using the User Engagement Scale Short From (UES-SF) [53]. This instrument consists of 4 subscales: *focused attention*, *perceived usability*, *esthetic appeal*, and *reward factor*. A total of 3 items measure each subscale.

The instrument to investigate *PE* of the breathing training consists of the following six items [54]: (1) *The breathing training facilitates relaxation*, (2) *The breathing training is pleasant to use*, (3) *It is easy to follow the breathing training instructions*, (4) *The breathing training effectively teaches how to breathe*, (5) *The breathing training is effective in reducing stress*, and (6) *The breathing training is effective in increasing attention to breath*. Each item was rated on a 5-point Likert scale (strongly disagree to strongly agree). To build the score for PE, we averaged the scores from all items. To construct the score for perceived relaxation effectiveness, we used the average of items 1 and 5.

Participants reported their *perceived breathing detection accuracy* with 2 independent items. The first one was adapted from the study by Efendic et al [55] and asked “How accurate is the breathing detection?” It was rated on a 7-point Likert scale (very inaccurate to very accurate). The second item asked “How much of your breathing did the breathing detection correctly detect?” Participants responded using a slider ranging from 0% to 100%. The questionnaires used in the study can be found in Multimedia Appendix 2.

We used 5 different smartphones in the study: *Samsung Galaxy S10e*, *OnePlus 6*, *Huawei P30 Lite*, *iPhone XR*, and *iPhone 11 Pro*. Each participant used only 1 smartphone, which was randomly assigned. All participants used Apple AirPods second generation [56] as headphones.

#### Procedure

After they signed the consent form at the start of the study, the participants received one of the smartphones with the stand-alone version of Breeze 2. The investigator then asked the participants to perform 2 breathing sessions with Breeze 2, one performed using headphones and the other without any additional hardware aside from the smartphone. Whether the participants started with or without headphones was randomly assigned. Each session was 3 minutes long. Before the first session, the investigator instructed the participants to read through the tutorial (Figure 1) and asked them to set the training duration and the breathing pattern to 3 minutes and 6 BPM, respectively. The investigator encouraged the participants to ask questions freely if the instructions provided in the app were not clear enough. We decided to allow such an additional explanation as assessing the quality of the tutorial was not a major objective of this study. After the first breathing session, the participants answered questions about their engagement (UES-SF) [53], the PE of the visualization [54], and the perceived accuracy of the breathing detection algorithm (adapted from the study by Efendic et al [55]) and provided their age and sex. Subsequently, they performed the second round of breathing training, after which they again answered the questions regarding the perceived accuracy of the breathing detection algorithm. If the participants wanted, they were allowed to interact with Breeze 2 for an additional 5 minutes, but this part was optional. Finally, the investigator encouraged participants to share feedback regarding Breeze 2 and the study. The sounds captured during breathing training were recorded for further offline analysis and future training data to refine the model.

#### Data Collection

Breeze 2 continuously monitors the breathing phase reference shown to the user during training and the breathing phases detected by the model used with and without the heuristic. This information is sampled every frame and, thus, usually results in 30 data points per second that are written to a log file. However, this number fluctuated depending on the smartphone’s computational power and the current scenery shown. Breeze 2 recorded and stored audio through the device’s microphone during training sessions. When the participants used headphones because of the study design, Breeze 2 recorded the audio with the headphones’ microphones. Breeze 2 tried to record with 44.1 kHz. However, operating system settings could overwrite this setting. In these cases, Breeze 2 recorded audio with at least 16 kHz, sufficient for the breathing detection model used. Breeze 2 similarly recorded the pre- and posttraining voice commands and yielded 2 separate recordings from the main breathing training recording. However, the voice commands were not further analyzed in this study.

After the data collection, 2 raters independently labeled the breathing training recordings as *exhalations* and *inhalations*. The raters did not manually label nonbreathing sounds. However, if a part of the recording was not assignable to an exhalation, an inhalation, or another sound, it was labeled as *unclear*. Unlabeled portions of the recording were then automatically labeled as nonbreathing sounds. A Cohen κ of 0.91 indicated near-perfect interrater reliability. Most mismatches came from slightly different label start and end times in the time-series data. Start or end time differences of >200 ms were manually inspected and merged, and others were merged by choosing the average of both raters. In case different class labels were assigned, either one of the raters’ labels was chosen for the corresponding segment or it was marked as unclear. We then transformed the labeled data into a data set following the same steps as the training data. The resulting data set consisted of 20,753, 10,459, and 19,265 samples for exhalation, inhalation, and nonbreathing sounds, respectively.

#### Statistical Analyses

For the collected data, we formulated the following hypotheses: (1) the engagement score is higher than the neutral score (neither agree nor disagree; hypothesis 1); (2) the PE is higher than the neutral score (neither agree nor disagree; hypothesis 2.1); (3) the perceived relaxation effectiveness is higher than the neutral score (neither agree nor disagree; hypothesis 2.2); (4) the balanced detection accuracy of the model alone is lower than the balanced exhalation detection accuracy, including the heuristic (hypothesis 3.1); (5) the balanced detection accuracy is lower for sounds captured by headphone microphones than by smartphone microphones (hypothesis 3.2); (6) there is a difference in balanced detection accuracy for female and male participants (hypothesis 3.3); and (7) the perceived detection accuracy correlates with the actual balanced breathing detection accuracy (model including the heuristic; hypothesis 4).

To ensure construct reliability, we calculated the McDonald ω [57] for all subscales of the UES-SF and the overall user engagement score (UES), PE, and perceived relaxation effectiveness. For all subsequent hypothesis tests, we used an α level of .05. To test hypotheses 1, 2.1, and 2.2, we conducted Wilcoxon signed-rank tests against the neutral score of 3.0 on the UES-SF, the PE, and the perceived relaxation effectiveness. To account for the familywise error rate of PE and perceived relaxation effectiveness, we applied the Bonferroni correction to adjust the *P* values. To gain more insight, we conducted Wilcoxon signed-rank tests for the 4 subscales of the UES-SF and applied the Bonferroni correction to adjust the *P* values to account for the familywise error rate. For hypotheses 3.1 to 3.3, we calculated the balanced detection accuracy of the model, including the heuristic and the model alone based on data from the log files aggregated with the labels of the audio recordings. We used balanced accuracy as the heuristic should increase exhalation sensitivity while decreasing the specificity. It should also affect the sensitivity and specificity measure for the other 2 classes.

Consequently, we used multi-class balanced accuracy [58] as the dependent variable for this analysis as it includes all classes’ specificity and sensitivity measures [59]. We then conducted a repeated-measure ANOVA with balanced accuracy as the dependent variable, the presence of the heuristic and the use of headphones as repeated-measure factors, and the participants’ sex as a between-subject factor. The latter was included to account for any potential breathing sound differences between men and women because of physiological differences [52]. A Shapiro-Wilk test [60] verified the normal distribution of the data for all 4 cells: heuristic (*W*=0.93; *P*=.07), headphone (*W*=0.97; *P*=.68), heuristic and headphone (*W*=0.94; *P*=.14), and neither (*W*=0.97; *P*=.55). We tested the assumption of homogeneity of variances for all the sex-based subgroups within the cells using the Brown-Forsythe test, which is a more robust Levene test [61] using medians instead of means to calculate the center of each group [62]. The assumptions of homogeneity of variance were met for heuristic (*F*_1,25_=1.75; *P*=.20), headphone (*F*_1,25_=0.07; *P*=.79), heuristic and headphone (*F*_1,25_=0.07; *P*=.80), and neither (*F*_1,25_=0.79; *P*=.38). The assumption of sphericity was met as the repeated measures had only 2 levels. To investigate hypothesis 4, we conducted Pearson correlation tests between the actual balanced breathing detection accuracy and the perceived detection accuracy items separately. This tested whether the found correlations differed from 0. We then scaled the responses from the response values to be between 0 and 1 (divided by the maximum allowed value for each item) and plotted them with the balanced breathing detection accuracy in 2 Bland-Altman plots [63] to investigate the tendencies of the differences.

#### Model Evaluation

We also investigated the detection performance of the model (excluding the heuristic) offline on the collected audio recordings. This was done for 2 reasons; first, to obtain detailed insights into the model’s detection performance without the heuristic used. Thereby, we obtained more information on the transferability of the model to potential other implementations where the heuristic would not be adequate. Second, this evaluation may serve as a baseline for future work as it was done in a standardized way offline on the collected breathing recordings. We considered the ROC curve of each class. Furthermore, we investigated the precision, recall/sensitivity, specificity, *F*_1_ score, and balanced accuracy (average of sensitivity and specificity) metrics. We included all these metrics to provide a complete picture of the model’s performance. Furthermore, we analyzed the detection performance for samples captured via smartphone and headphone microphones.

#### Data Exclusion

For the analyses regarding hypotheses 3.1 to 3.3, we excluded 10% (3/30) of the participants (3 women). One participant had technical problems with the headphones, which resulted in them performing the exercise twice without headphones. Another participant failed to disconnect the headphones, resulting in them performing the exercise twice with headphones. For the third participant, headphones could not capture any sound because of very silent breathing, whereas the smartphone microphone was able to capture some exhalations and missed most inhalations. We also excluded this third participant from the offline evaluation as the raters labeled most of the data from this participant as unclear. If we had included this participant, the analysis would have falsely shifted toward the hypotheses and arbitrarily favored the model’s performance in the offline evaluation.

However, the data of these participants were included for all the other tests as the participants were still able to complete the 2 breathing training sessions, although the third participant received very erroneous breathing feedback. For the latter, the model predicted 99.46% of the headphone session and 93.53% of the smartphone session as nonbreathing because most of the captured sound was completely silent.

### Ethics Approval

The Ethics Commission of the Swiss Federal Institute of Technology in Zurich (ID 2021-N-134) approved the study, and we pretested the study with 3 participants (1 woman).

## Results

### Checks for Reliability

We calculated reliability checks (Table 4) using the McDonald ω for the UES-SF and its subscales and for PE and perceived relaxation effectiveness (items 1 and 5 of the PE construct). The data from all these scales met the tests for normal distribution.

**Table 4:** Reliability tests for each survey construct.

Construct and subscale (number of items)			McDonald ω
**User Engagement Score Short Form (12)**			0.78
	Focused attention (3)	0.53	
	Perceived usability (3)	0.58	
	Esthetic appeal (3)	0.79	
	Reward factor (3)	0.82	
Perceived effectiveness (6)			0.58
Perceived relaxation effectiveness (2)			0.85

### Hypothesis Tests

#### User Engagement

A Wilcoxon test indicated that the UES was higher than the neutral response (mean 3.77, SD 0.43) for the participants (*W*=459; *P*<.001). The difference was also observed for all the subscales: focused attention (mean 3.22, SD 0.66; *W*=245; adjusted *P*=.15), perceived usability_rev_ (mean 3.90, SD 0.66; *W*=348; adjusted *P*<.001), esthetic appeal (mean 4.00, SD 0.547; *W*=406; adjusted *P*<.001), and reward factor (mean 3.97, SD 0.69; *W*=390; adjusted *P*<.001).

#### Effectiveness

The reported PE was higher than the neutral response (mean 4.08, SD 0.49), as shown by a Wilcoxon test (*W*=465; adjusted *P*<.001). In addition, for perceived relaxation effectiveness (mean 3.82, SD 0.95), a Wilcoxon test indicated a positive effect (*W*=358; adjusted *P*<.001).

#### Breathing Detection Performance

**Figure 9:**
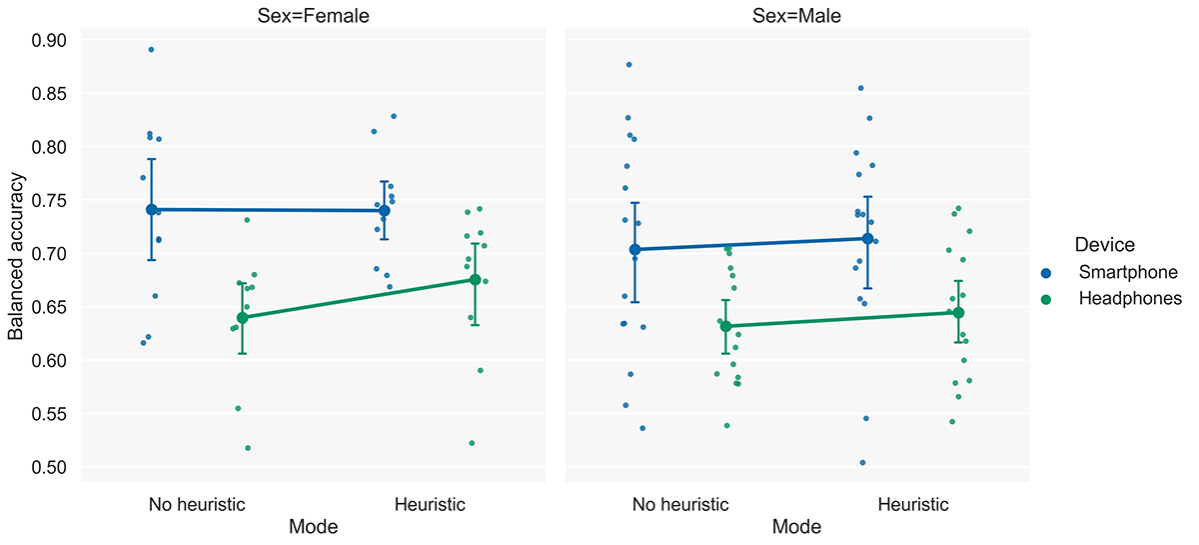
Marginal means plots illustrating the effects and interactions when different devices (smartphones and headphones) and model modes (no heuristic and heuristic) are used. Furthermore, the differences between female and male participants are also depicted.

A repeated-measure ANOVA indicated the presence of significant effects of headphone use (*F*_1,25_=17.61; *P*<.001) and use of the heuristic (*F*_1,25_=6.23; *P*=.02) on the detection performance of the model. The analysis did not indicate any interaction effects between the use of headphones and the use of the heuristic (*F*_1,25_=3.39; *P*=.08). Furthermore, no interaction effects of sex were found with headphone use (*F*_1,25_=0.11; *P*=.74), use of the heuristic (*F*_1,25_=0.25; *P*=.62), or both (*F*_1,25_=2.53; *P*=.12). In addition, no between-subject effects of sex were found (*F*_1,25_=1.38; *P*=.25). Figure 9 illustrates the estimated marginal means.

#### Perceived Breathing Detection Performance

**Figure 10:**
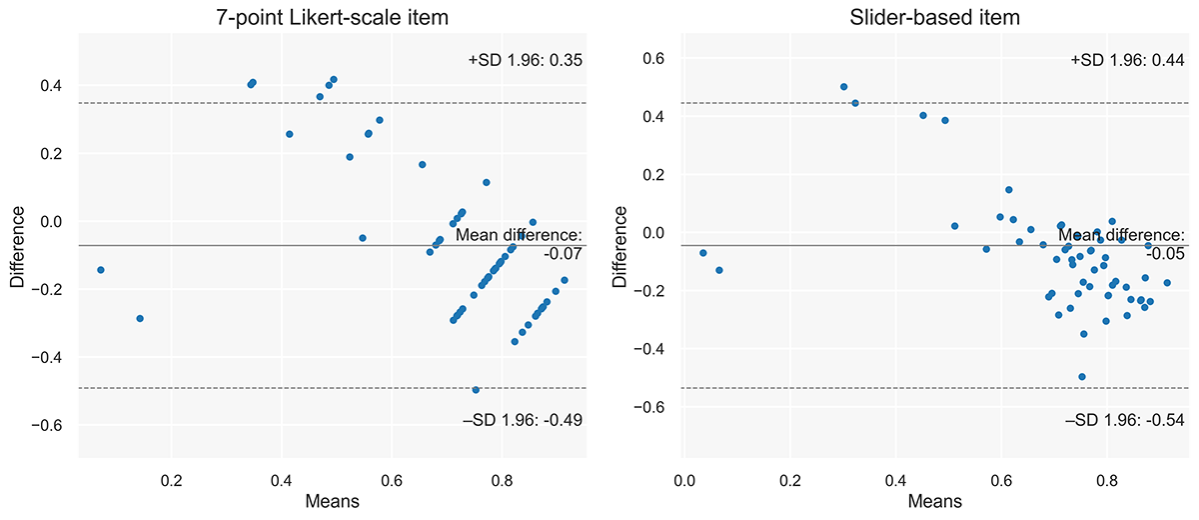
Bland-Altman plots for the 2 items measuring perceived breathing detection accuracy. Higher differences underestimate the actual detection accuracy, and lower values overestimate the detection accuracy. The limits of agreement are set to a 1.96 SD, which produces 95% CIs for the means of the differences.

The perceived breathing detection accuracy reported via a 7-point Likert scale (mean 5.17, SD 1.75) and 0 to 100 slider (mean 71.17, SD 28.68) showed some correlation with the actual performance of the breathing detector (mean 0.69, SD 0.08). The Likert scale showed a stronger correlation (*r*=0.51; *P*<.001) with the actual detection performance than the perceived accuracy reported via the slider (*r*=0.48; *P*<.001). Nevertheless, both correlations were significant. Bland-Altman plots (Figure 10) for both items showed that, when actual breathing detection accuracy was low, participants overestimated the accuracy. At the same time, they underestimated the accuracy when the actual detection accuracy was high.

### Offline Breathing Detection Model Evaluation

**Figure 11:**
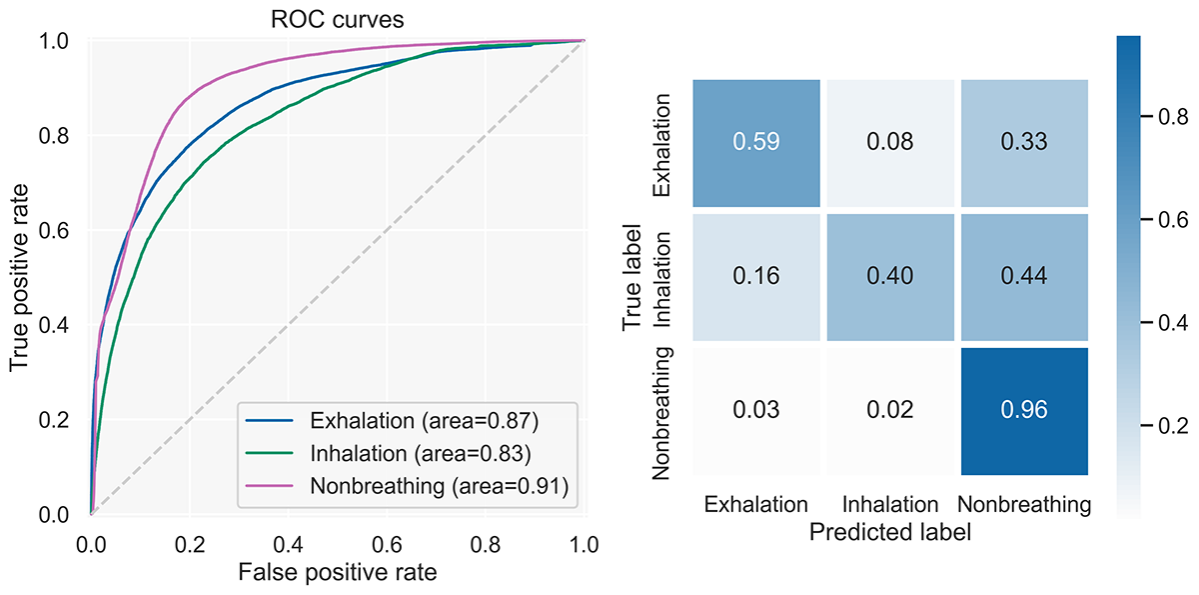
ROC curves (left) and confusion matrix (right) at the thresholds for breathing sounds that were used during the study. Both are calculated for the deployed model based on all data gathered throughout the breathing training sessions of the study participants. ROC: receiver operating characteristic.

The offline evaluation of the model (no heuristic) resulted in areas under the curve of 0.83, 0.87, and 0.91 for inhalation, exhalation, and nonbreathing sounds, respectively (Figure 11). The detailed results grouped by capturing device used on precision, recall/sensitivity, specificity, and balanced accuracy are shown in Table 5.

**Table 5:** Performance metrics of the model on the data captured during the study.

Class and device			Precision		Recall/sensitivity		Specificity		*F*_1_ score		Balanced accuracy
**Exhalation**											
	Combined	0.85		0.59		0.93		0.69		0.76	
	Smartphone	0.84		0.66		0.91		0.74		0.78	
	Headphones	0.86		0.51		0.94		0.64		0.73	
**Inhalation**											
	Combined	0.67		0.40		0.95		0.50		0.68	
	Smartphone	0.68		0.52		0.93		0.59		0.73	
	Headphones	0.65		0.26		0.97		0.37		0.61	
**Nonbreathing**											
	Combined	0.62		0.96		0.63		0.75		0.79	
	Smartphone	0.65		0.92		0.73		0.76		0.82	
	Headphones	0.59		0.99		0.53		0.74		0.76	
**All classes (average)**											
	Combined	0.71		0.65^a^		0.84		0.65		0.74^b^	
	Smartphone	0.72		0.70^a^		0.86		0.70		0.78^b^	
	Headphones	0.70		0.59^a^		0.81		0.59		0.70^b^	

^a^Corresponds to multi-class balanced accuracy according to Kelleher et al [64].

^b^Corresponds to multi-class balanced accuracy according to Urbanowicz and Moore [58].

## Discussion

### Principal Findings

Overall, Breeze 2 was well received, and all 30 participants could handle all aspects of it. Furthermore, all participants (30/30, 100%) successfully performed two 3-minute sessions of breathing training.

The participants perceived Breeze 2 as engaging according to the UES that differed significantly from the neutral response. Thus, our data support hypothesis 1. The in-depth analysis of the focused attention, perceived usability, esthetic appeal, and reward factor subscales revealed that participants particularly liked the esthetics and perceived reward factor of the experience, which were significantly higher than the neutral response. Interpretations of the focused attention and perceived usability scores were less conclusive than for the other 2 subscales owing to low reliability scores. Nevertheless, the average perceived usability was high and did differ significantly from the neutral response. However, focused attention was not significantly higher than the neutral response even though it had an average score with a positive tendency. This finding indicates that participants felt only moderately absorbed in the experience.

A feeling of absorption is important as this may lead to a flow state that helps people concentrate and perceive the task as rewarding and fun [65]. A possible solution for this could be to try out different modalities for breathing training such as virtual reality setups as such setups show promise for mindfulness exercises [24,66]. However, this would defeat the purpose of the objective regarding the intervention’s scalability. Another approach could be to introduce more dominant short-term feedback loops [65] during training to foster immersion as the overall reward factor of the experience already appears to be high. Nevertheless, such feedback loops need to be implemented with care as too strong and exciting loops may counter the targeted effects of the training (eg, relaxation).

Our data also supported hypotheses 2.1 and 2.2, as PE and perceived relaxation effectiveness were significantly higher than the neutral response. However, although the reliability score for perceived relaxation effectiveness was high, it was relatively low for PE. Thus, the scores for the latter should be interpreted with caution. We argue that this low reliability could be because the PE scale includes the perceived relaxation effectiveness scale and several other items asking about not equally perceived aspects of the breathing training. For example, a few participants (5/30, 17%) did not feel relaxed by the training but still thought it was easy to follow the instructions and directed their attention to their breathing. This is supported by the fact that the perceived relaxation effectiveness subscale yielded high reliability while having a lower mean than the overall effectiveness scale. Nevertheless, the analyses support hypotheses 2 and 3, meaning that the participants overall regarded Breeze 2 as effective in guiding their breathing and, most importantly, inducing a feeling of relaxation. The results are in line with prior work [34,54].

The analysis regarding the impact of the heuristic on detection performance showed that the heuristic brought a significant improvement to the overall detection performance, thus supporting hypothesis 3.1. The use of headphone microphones instead of the built-in microphone of the smartphones had an even larger but negative effect on detection performance. This even larger negative effect supports hypothesis 3.2. We argue that there are 2 reasons for this. First, the use of the smartphone microphone allows the model to detect exhalations through the generated airflow. The initial model evaluation has shown that this works better than acoustic detection. Second, modern Bluetooth headphones are optimized for speech and, thus, use filters to reduce noise in audio signals (eg, the Apple AirPods second generation used [56]). Breathing sounds are very close to noise (eg, white noise) and, thus, trigger these reduction algorithms.

Consequently, headphones may heavily suppress the breathing signal before the signal is passed to the model. How strongly these 2 reasons affect the observed negative effect remains unclear as data labeling did not differentiate between acoustically captured exhalations and exhalations captured through airflow. Regarding hypothesis 3.3, we did not observe any between-subject effects on detection performance based on the participants’ sex.

In addition, our findings support hypothesis 4 as perceived breathing detection accuracy significantly correlated with the actual detection performance. Thus, perceived breathing detection accuracy appears to help capture how clearly the feedback is perceived and how well the algorithm performs. We observed that participants over- and underestimated the detector’s performance when the actual performance was low and high, respectively. This over- and underestimation could indicate that the specific breathing feedback implementation in Breeze 2 gives users the sense of valid feedback even when the model performance is lacking. While conducting the study, we observed that participants felt more comfortable with the Likert-scale item than the slider-based item. Consequently, we plan to use the Likert-scale item in future studies to monitor perceived breathing detection accuracy in case changes need to be made to the feedback mechanism while Breeze 2 is deployed in the field.

Regarding the model without the heuristic, an apparent decline in detection performance was observed compared with the original test data set. The reasons for this are manifold. The breathing sound training and original test data sets were captured in the same setup [21]. This setup also differed considerably from the setup used in this study. In this study, the participants used Breeze 2 in a realistic setting for the first time by holding the device in their hands however was comfortable for them.

Consequently, the sound capturing was done in a much less regulated way. Furthermore, the training data used only a minimal number of devices shared between training and test data sets, whereas this study used smartphones and headphones unseen by the model. The headphones also pose a much more complex detection problem, as seen by the analysis regarding hypothesis 3.2. Our idea of a more complicated detection problem receives further support through the observation that all model performance metrics improve considerably for data only captured by smartphone microphones. This effect much more strongly influences the inhalation detection performance. We explain this through the observation that inhalations themselves are already very silent sounds and, thus, are already hard to detect. The attenuation applied by the headphones reinforces this problem even further.

Nevertheless, the model still performed reasonably well for exhalation sounds for both device types. The exhalation detection performance suffers, especially in sensitivity. However, the low sensitivity is less crucial as the model runs an inference up to 30 times per second, and the model yields high precision on exhalations. This reasoning is supported by the fact that participants overestimated the model’s performance in most sessions (44/60, 73%), even when the model did perform poorly. Consequently, the model appears to be already usable to enable interactivity in breathing training despite apparent weaknesses.

### Limitations and Future Work

Optimizing Breeze is an iterative process and, consequently, it comes with several limitations. The slide-based tutorial is not very engaging and does not yet provide adequate guidance on breathing training details. We plan to improve the tutorial by providing video-based instructions. In addition, we are considering implementing an interactive tutorial to ensure that users can perform the breathing training correctly and give them feedback right away before they embark on an actual training session. Aside from the still too basic tutorial, Breeze 2 does not yet provide an adequate mechanism to coach the users on choosing the suitable training duration and breathing pattern apart from labeling the different durations according to their level of expertise with breathing training. We plan to develop interactive tests that measure users’ capabilities and classify their level of experience (eg, based on the maximal time a person can inhale or exhale or on the user’s resonance frequency that maximizes the physiological response). Such tests would then allow us to offer some coaching to the user on which training parameters would fit their level. Finally, we have planned various studies to incorporate Breeze 2 as an intervention component in multicomponent interventions (eg, interventions aiming to prevent NCDs, reduce distress in patients with cancer, or reduce acute stress in a student population).

This study also has some limitations regarding the detection model and its performance. A total of 2 raters did the labeling independently, and disagreements were carefully handled in a subsequent process. Consequently, confidence is high that the labels are correct. Nevertheless, 1.51% of the recordings were labeled as unclear in the resulting data set. Unclear parts were not used for further analysis, potentially leading to a slight overestimation of the performance of breathing detection.

Furthermore, breathing can be very silent and, thus, may sometimes not be captured by microphones or may be actively suppressed by algorithms in the hardware used (eg, Bluetooth headphones). Therefore, the reported detection performance should be considered as the detection performance on breathing sounds that could be captured by the devices used. Furthermore, we trained the model on a minimal data set. Even though most participants perceived the model as performing well, there is room for improvement. With this study, we took the first step by collecting new data, which we will use to improve the model for future deployments.

### Conclusions

This paper presented Breeze 2, a new iteration of the gameful breathing training app Breeze. It consists of a slow-paced breathing training guided by gameful visualizations and uses breathing-based interactions. Furthermore, it allows users to choose training parameters consisting of training duration and breathing pattern. These features should improve long-term adherence to breathing training, support individuals in doing breathing training correctly, and help continuously increase training intensity. To gain insight into whether Breeze 2 is engaging and effective and into the performance of the breathing detection used, we conducted a laboratory study with 30 participants. Results show that most participants (28/30, 93%) perceived Breeze 2 as engaging and effective.

Furthermore, breathing detection performed sufficiently well for most participants’ sessions (50/60, 83%), as indicated by the perceived detection accuracy and actual detection accuracy. We attribute the exceptions to the combination of noise filtering done by the headphones and the very silent breathing of these participants, which was not audible in the recordings even to the raters conducting the labeling. We will use the collected breathing sounds to refine breathing detection, making it more stable and increasing its performance. Future work will use Breeze 2 as an intervention tool in various studies for the prevention and management of NCDs.

## Appendix

Multimedia Appendix 1 Screen recording of a breathing training session with Breeze 2.

Multimedia Appendix 2 Questionnaire used in the user study.

## Abbreviations

BPM: breaths per minute
HRV: heart rate variability
NCD: noncommunicable disease
PE: perceived effectiveness
ROC: receiver operating characteristic
UES: user engagement score
UES-SF: User Engagement Scale Short Form
YAMNet: Yet Another Mobile Network
