# Supplementary material for: Breathing as an Input Modality in a Gameful Breathing Training App (Breeze 2): Development and Evaluation Study
Source: JMIR Serious Games. 2022 Aug 16;10(3):e39186. doi: 10.2196/39186 (PMC9428773; doi:10.2196/39186)
Supplement: Multimedia Appendix 2 [file games_v10i3e39186_app2.pdf]

Dear participant

Thank you for participating in this study.

The study will take approximately 15 minutes of your time. You will perform two sessions of slow-paced breathing training assisted by the app Breeze and answer some questions regarding your experience and your agitation before and after the training.

Random 1: Please connect the headphones to the smartphone.

A1.

Please read through the tutorial in the app. For this, please take the provided smartphone and tap on the "Tutorial" button. Carefully read through the tutorial. If anything is unclear, you may ask the research team member present to clarify.

Please demonstrate to the research team member present how you would conduct the training to ensure that the tutorial has conveyed all the important information. Once the research team member has approved you may continue with this survey.

Please make sure that the exercise duration is set to 3 minutes and the breathing cycles per minute are set to 6 BPM. For this, click the button "Settings" and select the corresponding settings. The correct settings may be already set.

E1. Right now I feel...

	definitely not (1)	not (2)	not really (3)	a little (4)	very much (5)	extremely (6)
... Restless	☐	☐	☐	☐	☐	☐
... Composed	☐	☐	☐	☐	☐	☐
... Uneasy	☐	☐	☐	☐	☐	☐
... Relaxed	☐	☐	☐	☐	☐	☐
... Absolutely Calm	☐	☐	☐	☐	☐	☐

Please start the breathing training and follow along by pressing the "Start" button on the smartphone.

Once the training is complete you can continue this survey.

G1. Right now I feel...

	definitely not (1)	not (2)	not really (3)	a little (4)	very much (5)	extremely (6)
... Restless	☐	☐	☐	☐	☐	☐
... Composed	☐	☐	☐	☐	☐	☐
... Uneasy	☐	☐	☐	☐	☐	☐
... Relaxed	☐	☐	☐	☐	☐	☐
... Absolutely Calm	☐	☐	☐	☐	☐	☐

H1.

	strongly disagree (1)	disagree (2)	neither agree nor disagree (3)	agree (4)	strongly agree (5)
I lost myself in this experience.	☐	☐	☐	☐	☐
The time I spent using Breeze just slipped away.	☐	☐	☐	☐	☐
I was absorbed in this experience.	☐	☐	☐	☐	☐
I felt frustrated while using Breeze.	☐	☐	☐	☐	☐
I found Breeze confusing to use.	☐	☐	☐	☐	☐
Using Breeze was taxing.	☐	☐	☐	☐	☐
Breeze was attractive.	☐	☐	☐	☐	☐
Breeze was aesthetically appealing.	☐	☐	☐	☐	☐
Breeze appealed to my senses.	☐	☐	☐	☐	☐
Using Breeze was worthwhile.	☐	☐	☐	☐	☐
My experience was rewarding.	☐	☐	☐	☐	☐

	strongly disagree (1)	disagree (2)	neither agree nor disagree (3)	agree (4)	strongly agree (5)
I felt interested in this experience.	☐	☐	☐	☐	☐

I1.

	strongly disagree (1)	disagree (2)	neither agree nor disagree (3)	agree (4)	strongly agree (5)
The breathing training facilitates relaxation.	☐	☐	☐	☐	☐
The breathing training is pleasant to use.	☐	☐	☐	☐	☐
It is easy to follow the breathing training instructions.	☐	☐	☐	☐	☐
The breathing training effectively teaches how to breathe.	☐	☐	☐	☐	☐
The breathing training is effective in reducing stress.	☐	☐	☐	☐	☐
The breathing training is effective in increasing attention to breath.	☐	☐	☐	☐	☐

J1.

	very inaccurate (-3)	inaccurate (-2)	somewhat inaccurate (-1)	neutral (0)	somewhat accurate (1)	accurate (2)	very accurate (3)
How accurate is the breathing detection?	☐	☐	☐	☐	☐	☐	☐

J2. How much of your breathing did the breathing detection correctly detect?

☐☐☐☐☐☐☐☐☐☐

K1. What is your age?

☐☐☐☐☐☐☐☐☐☐

K2. What is your gender?

Female ☐

Male ☐

Non-binary ☐

Prefer not to say ☐

Random 2: Please connect the headphones to the smartphone.

Random 1: Please disconnect the headphones from the smartphone.

You will now do the training again.

If you have not returned to the start screen of the app, please do so by pressing the "Menu" button.

Please start the breathing training and follow along by pressing the "Start" button on the smartphone.

Once the training is complete you can continue this survey.

N1.

	very inaccurate (-3)	inaccurate (-2)	somewhat inaccurate (-1)	neutral (0)	somewhat accurate (1)	accurate (2)	very accurate (3)
How accurate is the breathing detection?	☐	☐	☐	☐	☐	☐	☐

N2. How much of your breathing did the breathing detection correctly detect?

☐☐☐☐☐☐☐☐☐☐

O1. You are now free to interact with the app however you like for up to 5 minutes. This is optional.

I'll take the opportunity to try out the app further.

Yes ☐

No ☐

Please use the app now however you like for up to 5 minutes. When you want to continue or latest after 5 minutes, please continue this survey.

Q1. **Do you have any feedback regarding the Breeze app you want to share?**

Q2. **Do you have any feedback regarding this study you want to share?**
